# Geospatial and multilevel clustering of zero-dose children in Kikwit, Democratic Republic of the Congo in 2022

**DOI:** 10.1371/journal.pgph.0002617

**Published:** 2024-02-29

**Authors:** Armand Malembe Mutwadi, Joule Ntwan Madinga, Veerle Vanlerberghe, Placide K. Mbala, Marianne A. B. van der Sande

**Affiliations:** 1 Department of Public Health, Institute of Tropical Medicine, Antwerp, Belgium; 2 Epidemiology and Global Health Department, National Institute of Biomedical Research, Kinshasa, Democratic Republic of the Congo; 3 Department of Community Health, Kinshasa School of Public Health, University of Kinshasa, Kinshasa, Democratic Republic of the Congo; 4 Global Public Health Department, Julius Center for Health Sciences and Primary Care, University Medical Center Utrecht, Utrecht University, Utrecht, Netherlands; University of Cape Town, SOUTH AFRICA

## Abstract

Zero-dose children remain highly vulnerable to vaccine-preventable diseases and can sustain transmission even in highly vaccinated populations. The WHO Immunization Agenda 2030 has prioritised reaching out to these children. We assessed the spatial distribution of zero-dose children together with the associated risk factors in a provincial capital in the Democratic Republic of Congo. A cross sectional survey was conducted in the city of Kikwit between September 28 and October 14, 2022. Data were collected both at household and health area level. QGIS and SATscan were used to describe and identify hotspots among zero-dose children, and a mixed effect logistic regression model was used to identify risk factors. Overall, 1,863 children aged 12–23 months were enrolled. Kikwit city had a 16.3% zero-dose prevalence, with significant variation between and within health zones. Two hotspots were identified through geospatial analysis, each spanning multiple health areas. Multilevel analysis revealed significant clustering at health area level and found six associated risk factors. These include the absence of home visits by community health workers (aOR = 1.90), living more than a kilometre from a health centre (aOR = 1.95), the mother’s lack of tetanus vaccination (aOR = 3.16), and inability to name a vaccine-preventable disease (aOR = 3.20). However, secondary (aOR = 0.56) or tertiary (aOR = 0.21) education of mothers/guardians and belonging to Bunda (aOR = 0.36) or Mbala (aOR = 0.52) ethnicity reduced the risk of zero-dose. We observed a high prevalence of zero-dose children with a heterogeneous spatial distribution of epidemiological importance. Due to sub-zonal diversity, a health zone approach to reduce zero-dose immunization appears very limited. Zero-dose prevalence was related to the community health workers’ home visit, to the distance of residence to a health centre and to household-level factors. Geospatial results could help in targeting priority health areas and communities for vaccination.

## Introduction

Zero-dose (ZD) children are those who have not received a single dose of Diphtheria Tetanus and Pertussis (DTP) vaccine as part of the routine vaccination schedule by the age of 12 months. There were an estimated 12.4 million ZD children in 2020, the majority living in sub-Saharan Africa or other conflict-affected areas [1, 2]. Pre-Covid-19 pandemic investigations indicated that nearly 50% of vaccine preventable deaths occurred among ZD children [3]. The World Health Organisation Immunization Agenda 2030 (IA2030) identified reaching out to ZD children as a strategic priority and has set a target of a 25% reduction in the number of ZD children by 2025 and 50% by 2030 [1, 4].

Global vaccination coverage has significantly increased since the WHO Expanded Programme on Immunization (EPI) in 1974 as a result of routine immunization (RI) and additional targeted campaigns. However, over the past ten years, coverage levels have decreased or stagnated in a number of countries, including the Democratic Republic of the Congo (DRC). Surveys between 2010–2019 in 92 countries recorded prevalences of ZD in children aged 12 to 23 months from 5.2% in upper-middle income countries to 11.1% in low-income countries, for a total pooled prevalence of 7.7% [5].

According to routine data from the DRC national EPI, the coverage of DTP1 vaccine was consistently above 90% in the last 3 years, and even surpassed 100% in 2022 [6]. However, the routine gathering of immunisation data poses challenges and has limitations in precisely assessing vaccination coverage, thereby undermining its dependability for decision-making in low- and middle-income countries (LMIC) [7]. Population-based vaccination surveys are commonly employed to gather coverage rates that accurately represent the actual situation. Based on household vaccination surveys conducted in 2020 and 2021 in DRC, there was a minor decline in DTP1 coverage rates, decreasing from 83.0% to 80.9% correspondingly, and a rise in the prevalence of ZD children from 17.0% to 19.1% [8, 9]. These rates are above the previous estimate for low-income countries. In 2021, it has also been described that there were wide variations between DRC provinces and between health zones. For example, the Mai-Ndombe province had a ZD prevalence of 41.8% while its nearest neighbour, the Kwilu had 10.3% [10].

A health zone (HZ) in DRC is a vast surface with 100,000 to 200,000 inhabitants having a secondary level hospital, and is subdivided in health areas, covering about 10.000 inhabitants having one health centre and various community-based health posts and sites. The health centre is a first-level health care facility with a minimum package of activities, including immunization service, which is provided at the centre and during outreach activities. A health centre serves a population within a maximum radius of action of 8 to 15 kilometres [11]. It is not routinely followed up if vaccination coverage is heterogeneous across health areas. This information could guide catch-up vaccination efforts to reduce ZD prevalence. The major factor contributing to disparities between vaccination coverage and susceptibility to vaccine-preventable diseases (VPDs) is the poor management of routine immunisation (RI) and vaccination campaigns [12, 13]. Spatial data are needed to identify geographically vulnerable populations beyond survey data which are identifying individual and household characteristic -based risk groups. Globally, geospatial analysis are increasingly used to create zonal-level health and demographic maps [14–17].

The aim of this study was to investigate the geospatial distribution and prevalence of ZD children by conducting a survey among children aged 12 to 23 months in all health areas within Kikwit city, DRC. The national EPI is interested in this specific age range due to its relevance because children in this age group should have received all routine vaccines according to the national childhood vaccination schedule for 2022, and for comparability purposes, as numerous studies and systematic reviews have focused on this age group [18–20]. We also set out to assess risk factors related to ZD status among these children at both household and health area levels taking into account the variation between health areas.

## Methods

### Study design and setting

**Fig 1:**
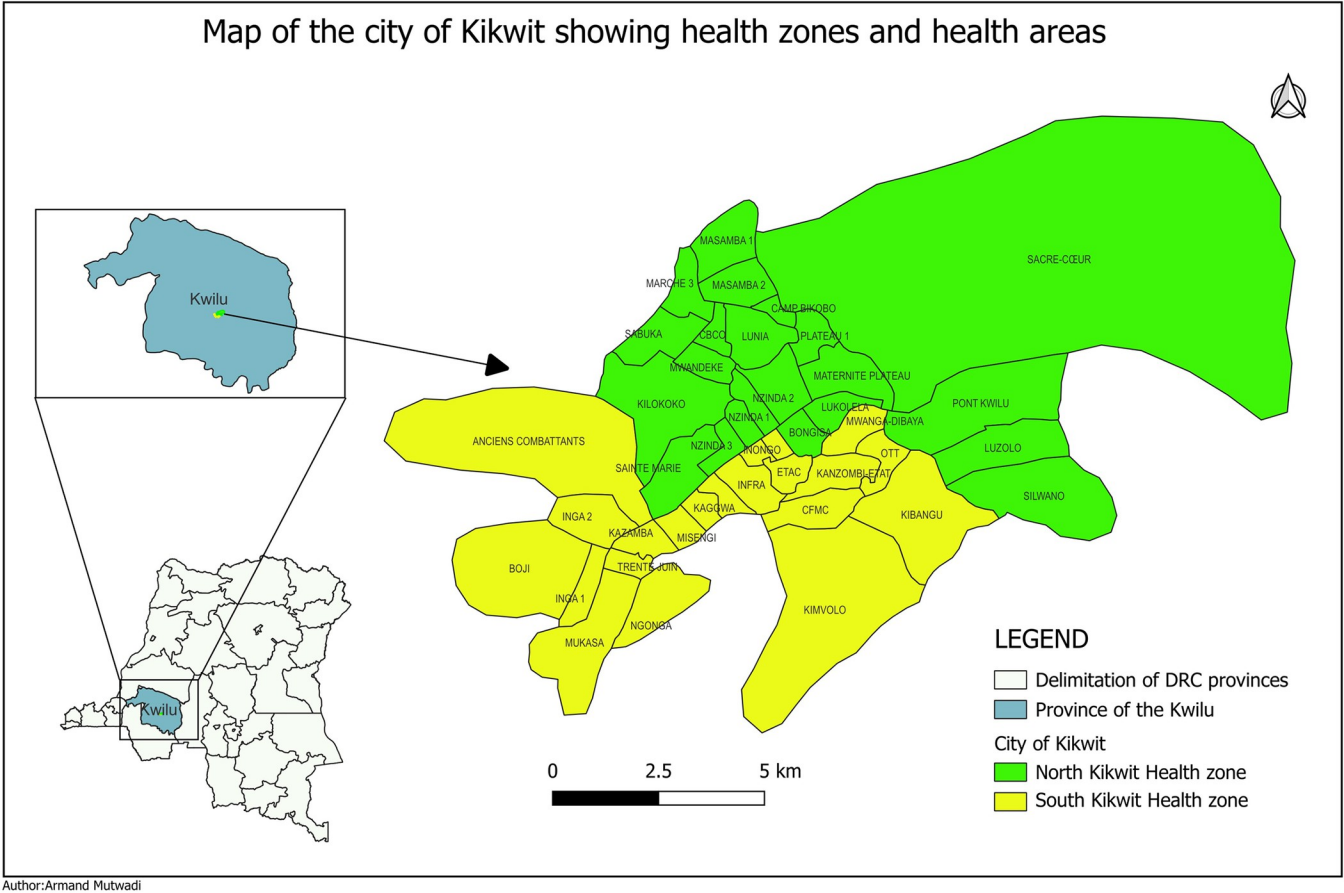
North Kikwit health zone with 25 health areas and South Kikwit health zone with 24 health areas, Kikwit city, province of Kwilu, DR Congo.

A cross sectional survey was conducted at the household and health area level between September 28th and October 14th, 2022, in the city of Kikwit, located in the Province of Kwilu at the western part of DRC. The city has two HZ, the North and the South HZ (***Fig 1***).

### Sampling

#### Household level

The survey targeted children aged 12 to 23 months at the moment of survey. Respondents were heads of household, and the mother or guardian of infant. Fathers were included in the study to provide the characteristics of household; in case of unavailability of father, this information was collected from the mother. Information on vaccine uptake was taken from the immunization card and if the card was not available, we relied on the mother’s or guardian’s recall. The study was powered to estimate a 50% ZD with 3% precision, taking into account a design effect of 2, and 10% non-response. Two stage cluster sampling was done. All the health areas within each HZ were included in the study. The North HZ has 25 health areas, and South 24. For the two zones (49 health areas = 49 clusters), the total sample size needed was 1764 children (900 children for the North and 864 for the South HZ) which means thirty-six children per cluster (health area, secondary sampling unit) was calculated.

In practical terms, we proceeded as follows:

■ First, in each cluster, 6 avenues in the urban setting (or 6 villages for the rural places) were selected by simple random sampling based on the list of avenues/villages provided.
■ Then, in each selected avenue/village, 6 households were selected based on a systematic random sampling technique.

#### Health area level

In each health area, the main health centre was visited to collect data on the health system, vaccination activities and demographic characteristics of its population. Hence, 49 health centres were included in this study. The respondent at health centre level was the head nurse or his/her deputy. Data were collected with an interviewer-based questionnaire.

### Data collection

The data was collected using “Kobocollect” on mobile devices [21]. All data, including GPS coordinates, were transmitted each day to a secure virtual server after verification for accuracy by the field supervisor.

### Outcomes

ZD child was defined as a child who had not received a single dose of Diphtheria Tetanus and Pertussis vaccine (DTP vaccine). According to the immunization schedule in DRC (***Table 1***), the first DTP should be administered at 6 weeks after birth, the second one at 10 weeks and the third, and last one at 14 weeks [22]. Furthermore, data were collected for possible risk factors as reported elsewhere (such as age, sex, migrant status, marital status, ethnicity, religion, mother’s profession, mother’s education, antenatal care attendance, previous maternal vaccination such as tetanus vaccine during pregnancy (maternal recall), name of at least one VPD, father’s profession, community health workers’ home visit, distance between household and the health centre) and at health area, on the availability of a refrigerator in the health facility, vaccine stockout during the last 12 months at health facility level, and accessibility of the health facility [23–32].

**Table 1:** The routine vaccination schedule in DRC [22].

Vaccine	Age of administration
BCG and OPV 0	at birth
OPV 1 and Penta 1 (DTP1) and Rota 1 and PCV13 1	at 6 weeks
OPV 2 and Penta 2 (DTP2) and Rota 2 and PCV13 2	at 10 weeks
OPV 3 and Penta 3 (DTP3) and Rota 3 and PCV13 3, IPV	at 14 weeks
MCV1 and Yellow fever vaccine	at 9 months

BCG, Bacile Calmette-Guerin, OPV, Oral Poliomyelitis Vaccine, DTP, Diphtheria Tetanus Pertussis, MCV, Measles Containing Vaccine.

### Statistical analyses

Completed questionnaire databases were transferred to IBM SPSS statistics version-27 and R studio 2023.03.0 Build 386 software for analysis.

Categorical variables were summarized as frequencies and proportions, continuous variables as mean with standard deviation (SD) or median with interquartile range, depending on the normality of distribution. The distance between household and the main health centre of the health area was computed using geographical coordinates with QGIS. The ZD prevalence was assessed separately by source of information (vaccination card and mother recall), and with both sources pooled.

The association between dependent and independent variables was determined using crude and adjusted odds ratios (OR) with 95% confidence intervals (95% CI). We have applied a Bonferroni correction, considering a p-value of 0.05 divided by 6 (number of comparisons at the final model) as statistically significant. We used two-level mixed-effect logistic regression modelling to simultaneously investigate health area and household level factors associated with the ZD children, allowing between health area variation.

Three multilevel logistic regression models were fitted. Model 1 was an empty model that contained only health area-specific random effects to model between health area variation with ZD children. Model 2 included the health area-specific random effects and the respondent characteristics, Model 3 included health area-specific random effects, respondent characteristics and the health area level characteristics.

From the bivariate regression, all variables with a p-value <0.25 were included in the multivariate analysis [33]. The final model was selected throughout a backward stepwise approach, based on the smallest Akaike information criterion (AIC). Multicollinearity among independent variables was checked by estimating the variance inflation factor. The “ANC visit” variable was removed from the multivariate analysis because it was constant to “mother receiving tetanus vaccine during the last pregnancy” variable. Intra-Class Correlation (ICC) and proportional change in variance (PCV) were used to quantify the magnitude of the effect of health area itself on ZD children [34]. We evaluated the accuracy of the final model using the receiver operating characteristic (ROC) by measuring the area under the curve (AUC).

### Spatial analysis

The household’s geographic locations were subjected to a spatial point patterns analysis [35]. All enrolled households were connected to latitude and longitude data. The population density data was downloaded from GRID3 DRC Gridded Population Estimates [36]. An exploratory analysis using the Ripley’s K function [37]was performed to determine whether ZD children tended to occur close to other ZD children [38]. The maximum and minimum values for the x and y coordinates were used to construct a grid containing the coordinates of ZD children [39]. The K function was then transformed into an L function which is easier to interpret visually [37]. The analysis was done in R Studio version *2023*.*03*.*0 built 386* for Windows using the *Spatstat* package.

Spatial clusters (hotspots) were identified using the spatial scan statistic in the SaTScan software [40]. A circular window shape was used with a maximum spatial cluster size of 50% of the population at risk. A likelihood ratio test is applied to each window, which is considered as a potential cluster, to test the null hypothesis of absolute spatial randomness against the alternative hypothesis that there is an elevated risk within the window as compared to outside the window. The inference was done by using Monte Carlo simulations where replications of the data set under the null hypothesis were generated. A Bernoulli distribution was used as the probability model and a cluster was significant if it had a maximum likelihood ratio higher than the maximum likelihood ratio from the most likely cluster generated in the random data set [41]. The level of significance was set at p = 0.05. All statistically significant clusters were mapped on to the study area using QGIS version 3.30.3.

### Missing data

Five children out of 1,868 enrolled (0.3%) did not have DTP and covariate information and were excluded from analysis.

### Ethical approval

Data collectors ensured that participants understood the purpose of the study and formal written informed consent was obtained from the parent/guardian at household level and from the head/deputy nurse at health facility level prior to participation in the study. The study protocol was approved by both the Institute of Tropical Medicine, Antwerp IRB board (IRB Approval letter FRM-1257v3.0) and the Kinshasa School of Public Health Ethics Committee (Approval N° ESP/CE/129/2022).

## Results

### Characteristics of households, mothers/guardians and children

Overall, 1,863 children aged 12 to 23 months were included in the analysis. There were 210 (11.3%) children in a migrant or returnee household; 33.1% had vaccination cards; and almost 67% of mothers/guardians declared to have not received visit of CHWs the three last months (***Table 2***). Nearly 16% of mothers/guardians were not able to quote at least one VPD (***Table 3***). The median distance between households and the main health centre inside a health area was 467 meters, with a minimum of 2.81 meters and a maximum of 4693.3 meters.

**Table 2:** Household characteristics, Kikwit city, DRC, 2022 (n = 1863).

Household characteristics	Total enrolled n (%)
**Health zone**	
North Kikwit	956(51.3)
South Kikwit	907(48.7)
**CHWs visit in past three months**	
Yes	672(36.1)
No	1191(63.9)
**Setting**	
Urban	1791(96.1)
Rural	72(3.9)
**Migrant status**	
Resident	1653(88.7)
Immigrant/returnee	210(11.3)
**Possession of a vaccination card**	
Yes	616(33.1)
No	1247(66.9)
**Marital status**	
Bachelor	29(1.6)
Married/common-law	1727(92.7)
Divorced/Separated	42(2.3)
Widower/widow	65(3.5)
**Religion**	
Catholic	470(25.2)
Christian	1093(58.7)
Don’t pray	107(5.7)
Kimbanguism/Black Church	77(4.1)
Muslim	28(1.5)
other religion	88(4.7)
**Ethnical group**	
Bunda	252(13.5)
Luba	86(4.6)
Mbala	429(23.0)
Pende	433(23.2)
Yansi	303(16.3)
Other	360(19.3)
**Household head profession**	
Farmer/breeder/fisherman	145(7.8)
Work for government	497(26.7)
Trader	248(13.3)
Unemployed person	230(12.3)
Private employee	634(34.0)
Other	109(5.9)
**Distance between household and health centre within the health area**	
Up to 1km	1447(77.6)
More than 1Km	416(22.3)

CHWs, Community Healthcare Workers

Returnee: autochthonous people who moved to another province for at least a year and had returned to the city for less than one year at the moment of the survey.

**Table 3:** Children and mother/ guardian characteristics, Kikwit city, DRC, 2022 (n = 1863).

Child and Mother/Guardian characteristic	Total enrolled n (%)
**Child sex**	
Female	824(44.2)
Male	1039(55.8)
**Child Nutrition status**	
BP </ = 11.5cm	40(2.1)
BP 11.6–12.5cm	329(17.7)
BP>12.5cm	1494(80.2)
**Relationship to the child**	
Guardian	222(11.9)
Mother	1641(88.1)
**Mother/Guardian age category**	
Less than 24 yrs.	509(27.3)
25–34 yrs.	933 (50.1)
35 yrs. Or more	421 (22.6)
**Mother/Guardian’s Education**	
Never/Primary	334(17.6)
Secondary	1395(75.2)
Tertiary	134(7.2)
**Mother/Guardian’s profession**	
Farmer/breeder	108(5.8)
Unemployed/Housewife	1181(63.4)
Work for government	182(9.7)
Worker/shopkeeper	269(14.4)
Other	123(6.6)
**Name at least one VPD**	
Yes	1568(84.2)
No	295(15.8)
**ANC visit**	
Yes	1779(95.5)
No	43(2.3)
Don’t know	41(2.2)
**Received tetanus vaccine during last pregnancy**	
Yes	1661(89.2)
No	118(6.3)
Not Applicable	84(4.5)

BP, Brachial Perimeter, VPD, Vaccine Preventable Disease, ANC, AnteNatal Consultation

### Health areas characteristics

**Fig 2:**
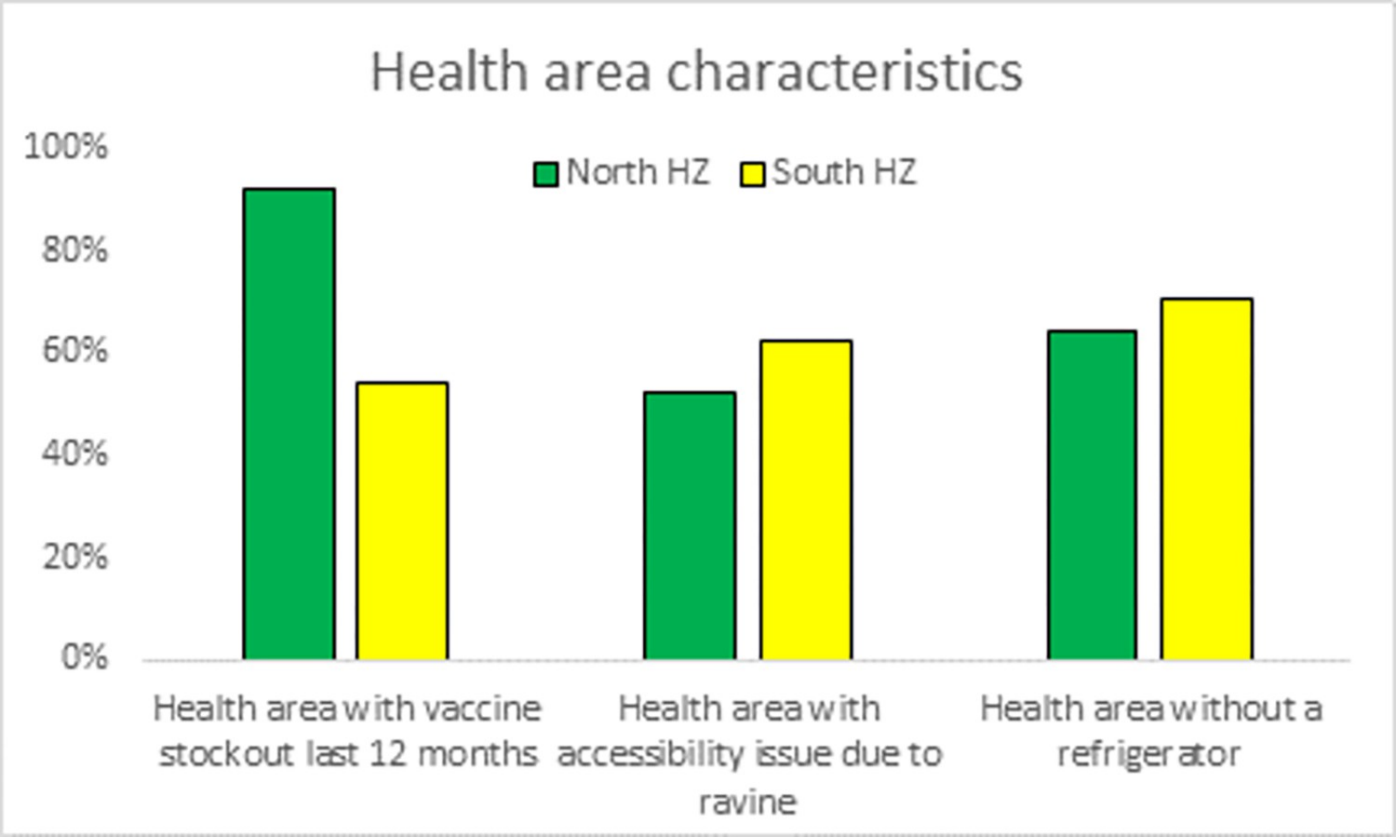
Health area characteristics, Kikwit city, DRC, 2022 (N = 49 health areas).

92% of health areas in the North Kikwit HZ reported stockout during the last 12 months while in the South HZ, 54.2% of health areas were affected by a stockout (p = 0.007). In both North and South HZ some parts were inaccessible due to ravines (p = 0.65). 70% of the health areas had refrigerators in the South and 64% in the North (p = 0.61). (***Fig 2***).

### Prevalence of zero-dose children

Overall, 616/1863 (33.1%) of households presented vaccination cards. For the remainder 1247 households, DTP vaccination status was based on maternal recall. Based on the vaccination card (n = 616), the prevalence of ZD was 3.5% (13/371) in the North HZ and 4.9% (12/245) in the South and based on maternal recall (n = 1247), this prevalence was 17.9% (105/585) in the North and 26.1% (173/662) in the South.

**Fig 3:**
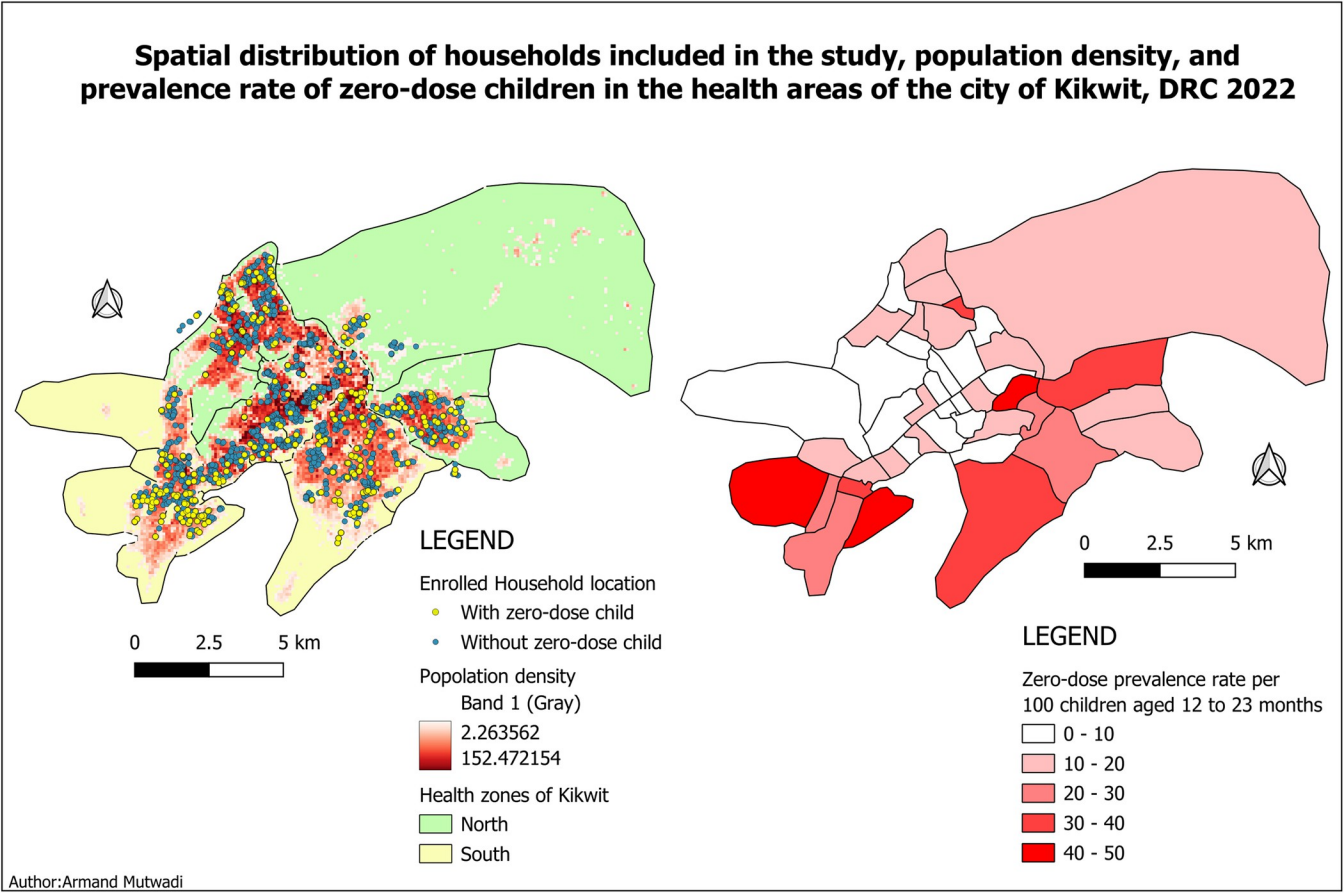
Spatial distribution of households enrolled the study, population density, and prevalence of ZD children at health area level considering both card of vaccination and maternal recall in Kikwit, 2022.

Overall, 303/1863 children (16.3%) were considered as ZD considering both vaccination card and mother recall, less in the North 12.3% (118/956) than in the South HZ 20.4% (185/907) (p <0.001). Significant variation in prevalence was found between health areas (p<0.001 for both HZ). In the North HZ, three health areas had a ZD prevalence equal to 0, and the highest prevalence was 33.3%. In the South HZ, one health area had a ZD prevalence equal to 0, while three health areas had a prevalence > 40%. (***Fig 3***).

### Geospatial distribution of ZD children

**Fig 4:**
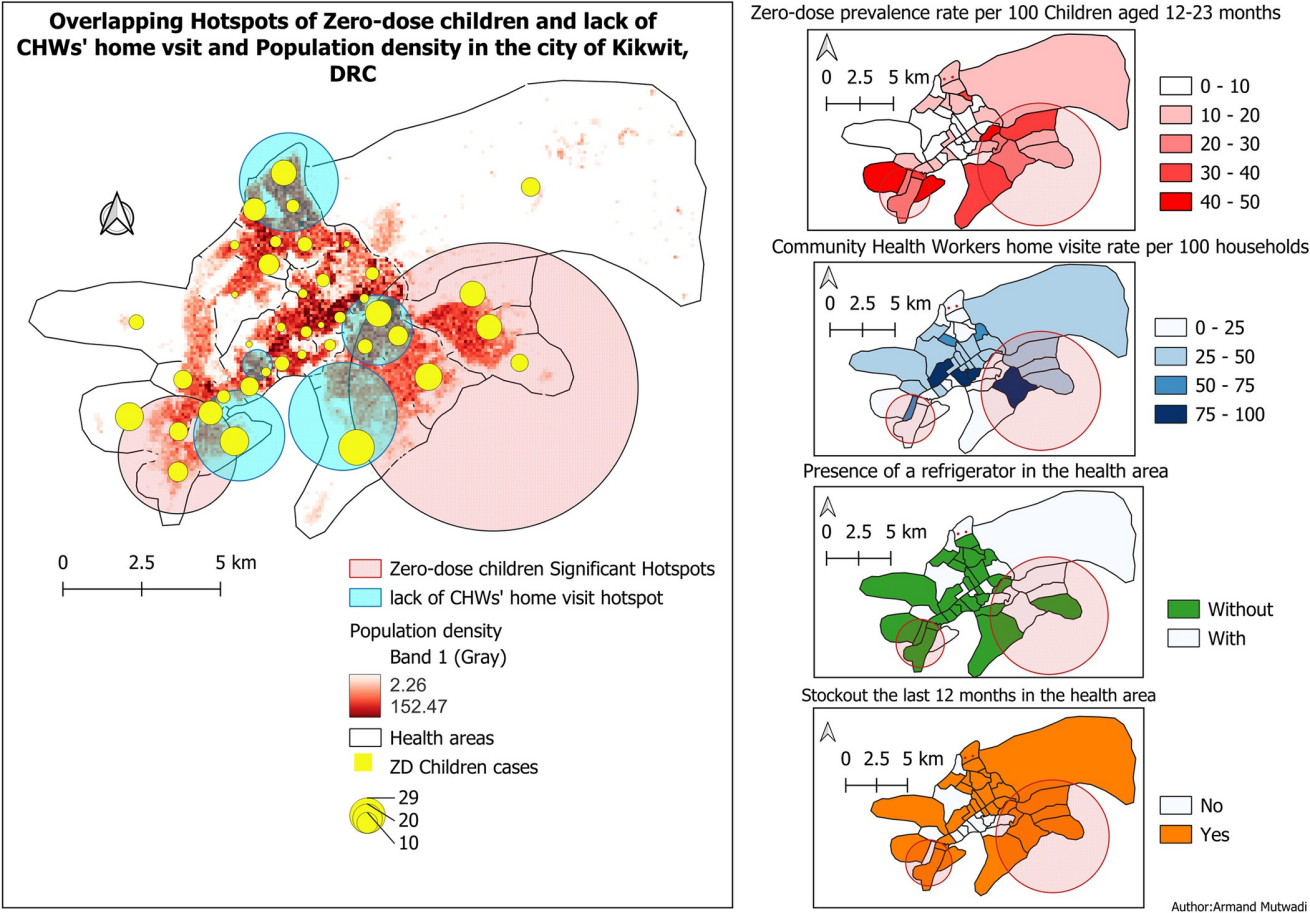
Hotspot analysis, population density, cases of ZD children in the health areas and the distribution of the prevalence of ZD children, CHWs home visit rate and the possession of refrigerator, Kikwit City, DRC, 2022.

Geographical point pattern analysis revealed a spatial clustering with two Hotspots of ZD children in Kikwit city. Hotspot 1 was restricted in the South HZ and had a relative risk of 2.48 of being zero-dose. Hotspot 2 covered six health areas in the South HZ and four in the North HZ and had a relative risk of 1.77 (***Table 4***) (***Fig 4***)

**Table 4:** Zero-dose hotspots features in Kikwit city, DRC, 2022 (n = 1863).

Features	Hotspot 1	Hotspot 2
Number of sampled households living in the hotspot	185	459
Number of ZD cases	65	111
Percent ZD cases in area	35.1	24.2
Expected Zero-dose cases	30.09	74.65
Relative risk	2.48	1.77
P-value	<0.001	0.004

Children living in these hotspots have a high risk of being ZD, whereby we observed that this risk was higher in hotspot 1 than in hotspot 2 (prevalence difference p-value 0.036). The expected number of ZD cases was based on the overall prevalence (16.3%).

There is an overlap between of Hotspots between ZD children and the lack of CHWs’ home visit. Health areas with a high prevalence of ZD children were located more on the outskirts of the city and those with a low prevalence were located more in the middle and west of the city. The CHWs home visit rate was less than 25 per 100 households in 4/5 health areas in the hotspot 1. Four health areas out of five (80%) lacked a refrigerator in the hotspot 1, and four out of twelve (33.3%) in the hotspot 2. Ten health areas (83.6%) in the hotspot 2 had stockout during the previous twelve months, while one (20%) in the hotspot 1.

### Risk factors of ZD children

In multivariate analysis, six variables were associated with ZD prevalence. The absence of visits from CHWs (aOR = 1.90), household located more than 1 kilometre from a health centrer (aOR = 1.95), not receiving maternal tetanus vaccination during the previous pregnancy (aOR = 3.16), and mothers/guardians unable to name at least one VPD (aOR = 3.20) significantly increased the risk of ZD. On the other hand, children of the *Bunda and Mbala* ethnical groups (respectively, aOR = 0.36 and 0.52) and those from mother/guardians with secondary (aOR = 0.56) or university (aOR = 0.21) level of education were at lower risk for ZD (***Table 5***). Vaccine stockout, lack of a refrigerator and inaccessibility of some part of the health area were not significantly associated with ZD prevalence.

**Table 5:** Zero-dose prevalence according to main characteristics and associated risk factors in Kikwit City, DRC, 2022.

Characteristics	Zero-dose children prevalence, % (n)	Bivariate analysis			Multivariate analysis (final model)		
		crude OR	95% CI	p-value	Adjusted OR	95% CI	p-value
**Migration status **							
Resident	15.1% (249)	1					
Migrant/Returnee	25.7% (54)	1.95	(1.39–2.74)	<0.001			
**CHW visit during the past 3 months **							
Yes	9.2% (62)	1			1		
No	20.2% (241)	2.5	(1.85–3.36)	<0.001	1.9	(1.28–2.81)	0.001*
**Distance, household to PHC within a health area **							
Up to 1km	15.7% (227)	1			1		
More than 1km	18.3% (76)	1.2	(0.9–1.60)	0.209	1.95	(1.26–3.03)	0.003*
**Ethnical group **							
Pende	21.7% (94)	1			1		
Bunda	8.7% (22)	0.34	(0.21–0.57)	<0.001	0.36	(0.20–0.66)	0.001*
Luba	25.6% (22)	1.24	(0.73–2.12)	0.432	1.12	(0.56–2.23)	0.757
Mbala	13.1% (56)	0.54	(0.38–0.78)	0.001	0.52	(0.33–0.81)	0.004*
Yansi	14.5% (44)	0.61	(0.41–0.91)	0.015	0.63	(0.39–1.01)	0.057
Others	18.1% (65)	0.79	(0.56–1.13)	0.201	0.82	(0.53–1.27)	0.366
**Religion **							
Catholic	12.1% (57)	1					
Christian	15.2% (166)	1.3	(0.94–1.79)	0.113			
Kimbanguist/black church	31.2% (24)	3.28	(1.88–5.72)	<0.001			
others	17.9% (5)	1.58	(0.58–4.31)	0.376			
Don’t pray	22.4% (24)	2.1	(1.23–3.57)	0.006			
Muslim	30.7% (27)	3.21	(1.89–5.45)	<0.001			
**Marital status **							
Married/common-law	16.1% (278)	1					
Bachelor	20.7% (6)	1.36	(0.55–3.37)	0.507			
Divorced/Separated	28.6% (12)	2.08	(1.05–4.12)	0.035			
Widower/widow	10.8% (7)	0.63	(0.28–1.39)	0.253			
**Profession **							
Farmer/breeder/fisherman	20% (29)	1					
Unemployed person	18.3% (42)	0.89	(0.53–1.51)	0.676			
Trader	18.5% (46)	0.91	(0.54–1.53)	0.724			
Work for government	9.1% (45)	0.4	(0.24–0.66)	<0.001			
Private employee	19.4% (123)	0.96	(0.61–1.51)	0.870			
Others	16.5% (18)	0.79	(0.41–1.51)	0.479			
**Name at least one VPD **							
Yes	12.2% (192)	1			1		
No	37.6% (111)	4.32	(3.27–5.72)	<0.001	3.2	(2.23–4.61)	<0.001*
**Received tetanus vaccine during the last pregnancy **							
Yes	13.0% (216)	1			1		
No	37.3% (44)	3.98	(2.67–5.93)	<0.001	3.16	(2.01–4.96)	<0.001*
**Mother’s/Guardian’s education **							
Never/primary	26.6% (89)	1			1		
Secondary	14.9% (208)	0.48	(0.36–0.64)	<0.001	0.56	(0.39–0.79)	0.001*
Tertiary	4.5% (6)	0.13	(0.05–0.30)	<0.001	0.21	(0.08–0.52)	0.001*
**Mother’s/Guardian’s age range **							
Less than 24 yrs.	20.4% (104)	1					
25–34 yrs.	15.3% (143)	0.7	(0.53–0.93)	0.014			
35 yrs. and more	13.3% (56)	0.6	(0.42–0.85)	0.004			
**Health area with some part inaccessible **							
No	17.0% (181)	1					
Yes	15.3% (122)	0.88	(0.69–1.14)	0.333			
**Refrigerator in health area **							
Yes	15.4% (193)	1					
No	17.9% (110)	1.2	(0.93–1.55)	0.169			
**Stockout in the past 12 months **							
No	11.4% (55)	1					
Yes	17.9% (248)	1.69	(1.24–2.32)	0.001			

CHWs, Community Healthcare Workers, PHC, Public Health Centre, VPD, Vaccine Preventable Disease

The ROC showed an area under the curve (AUC), of 83.9% (95%CI 81.5% - 86.2%), indicating the model was adequate in differentiating those not having received a single dose of DTP vaccine from those receiving it. Details regarding health area-level variance of the multilevel logistic models predicting zero-dose children and the random effect results are included in the next section.

### Random effect results

After considering both household and health area-level covariates (Model 3), health area-level variability was reduced from 49.3% to 29.2% (***Table 6***). The model also showed that 40.7% of health area-level variance on zero-dose was explained by the combined factors at both the household and health area levels.

**Table 6:** Health area level variance of the multilevel logistic models predicting zero-dose children in Kikwit city, DRC, 2022.

Random Effect	Model 1	Model 2	Model 3 (final)
Health area level variance	3.197	1.346	1.356
ICC (%)	49.3%	29.0%	29.2%
PCV (%)	Reference		40.7%
Model fitness statistics (AIC)	9168.4	9439.2	9332.5

ICC intra-class correlation, PCV proportional change in variance, AIC Akaike Information Criteria.

Although model 1 has the smallest AIC, this model included only the health area random effect, and no fixed effect was considered.

## Discussion

We found a zero-dose children prevalence of 16.3% in Kikwit city. This finding is higher than in earlier studies of LMICs and the province of Kwilu, to which the city belongs [5, 10] but less than the prevalence throughout the country of 19.1% [10, 25]. However, we found substantial differences in the prevalence between and within the two HZ of the Kikwit city. The South HZ was more affected by ZD children then the North HZ.

Global health experts have recently focused on pinpointing and targeting VPD-risk populations geographically. This helps identify underserved communities and facilitates more precise interventions and resource allocation [42]. Specific risk factors related to individuals/ households might be useful in identifying at-risk groups. However, it can be challenging to consider these characteristics when they are spread out over the geographical area being studied, particularly during vaccination campaigns. Our study demonstrates and sheds light on the existence of ‘geographical’ pocket and communities of ZD children within HZs in the city of Kikwit. These results provide evidence to decision-makers to develop targeted strategies where more effort is needed. We have identified two major hotspots for ZD children in the city, which were mainly located in the South HZ, with one overlapping some health areas in the North HZ. For a child located in this geographical area, the risk of not being vaccinated against DTP is twice as high compared to other parts of the city. As DTP is administered together with Haemophilus influenzae type B and Hepatitis B in the pentavalent vaccine in DRC, the risk of not being vaccinated against these pathogens is similar as ZD. Well beyond the timing of these vaccines, this child also has a high risk of not receiving measles and yellow fever vaccine, even while these are routinely given at a later age [22].

The Immunization Agenda 2030 aims to significantly reduce (50%) the number of ZD children by 2030 by achieving high and equitable coverage levels [1, 4]. The process of achieving this goal can be made easier with the use of geospatial and multilevel analysis.

In this study, within a single HZ, the distribution of ZD children was very heterogeneous, varying significantly between health areas (sub-zonal level). Thus, having information on the prevalence of ZD at the HZ level only is of limited value to target interventions, as there can be substantial heterogeneity between health areas. For instance, the zero-dose prevalence in the South HZ was 20.4%; while at the sub-zonal level, there is one health area with a ZD prevalence equal to zero and another health area with a ZD prevalence of more than 40%, so clearly the two communities do not have the same priority for intervention. To achieve equitable coverage levels, it is necessary that information on the level of coverage is detailed down to the sub-zonal level (health area-level).

The geospatial analysis also revealed that health areas located on the outskirts of the city were more affected by ZD children than those located in the middle of the city, and the middle of the city has a higher population density than the outskirts, which is in line with another study demonstrating that ZD children are more likely residing in missed communities that are difficult to access, or that receive less attention from political and health decision-makers [1]. The geospatial analysis could help not only in prioritizing missed communities but also in monitoring the resource allocation. 80% of health areas lacked a refrigerator and 83.3.6% had stockout during the previous twelve months, in the first hotspot. The geospatial approach has the potential of enhancing access to not just immunization activities, but also other primary health services missed by these communities.

We observed a substantial clustering of ZD prevalence within Kikwit city using multilevel analysis, with an Intra-class correlation of 29.2% (> conventional threshold of 5%) [43]. This suggests that health area-level factors account for 29.2% of the variance of the prevalence of ZD children in the city (after controlling for household-level factors), which is in line with previous studies in this field [29, 44], even though we did not find a significant association with vaccine stockout, absence of a refrigerator or accessibility problems in the health area. However, this study revealed a significant association between ZD prevalence and the CHW home visit and the distance between households and health centre which can be considered proxies of the general health system functioning. This highlights the need for interventions that target not only household or individual vaccination related factors, but also factors at community/health area level, such as community mobilization by CHWs and bringing the vaccination services closer to the households. The absence of CHWs’ home visits nearly doubled the risk of being zero-dose. CHWs cannot replace qualified medical professionals, but they are vital to the community and can increase service uptake. CHW programmes in deprived regions provide promotive, preventive care and patient referral services, helping to improve population health and advance universal health coverage [26, 27, 45]. However, it is unlikely that CHWs will perform this task on a volunteer basis given the increasing time demands on them. The possibility of paying them for their time and supporting their transportation expenses should be taken into account when defining nation-wide health policies.

Households located more than one kilometre from the health centre were associated with twice the risk of having ZD children compared with those located less than one kilometre away. According to the operating standards for a health centre in the DRC, the health centre serves a population within a maximum radius of action of 8 to 15 km [11] This result highlights the need to have several locations offering the immunization service within a health area and emphasises the importance of outreach in the delivery of vaccination to children.

Our findings showed that children of mothers/guardians with at least secondary education were less likely to be zero-dose than those with no schooling or primary education. Mothers/guardians who couldn’t name a single VPD had a threefold risk of having a ZD child compared to those who named at least one VPD. Several studies have linked maternal education to immunization rates [25, 28, 32, 46, 47]. Education of mothers positively impacts children’s health by providing good information and influencing beliefs, perceptions, and practices that unintentionally harm children’s health. Education brings knowledge, eliminates ignorance, and increases understanding of diseases. The fact that some mothers couldn’t name one VPD showed that the immunization information was inadequate for potential stakeholders in the area.

Mothers who did not receive a tetanus vaccine during pregnancy were more likely to have a ZD child than those who did. It exhibits similarities to the occurrences observed in India [48]. ANC informs women about vaccination [28–31], but agreeing to be vaccinated is a separate concept. During ANC sessions, health education is provided to encourage women to get vaccinated. CHWs play a crucial role in providing health education within the community. They possess the ability to effectively tackle cultural obstacles (such as home birth) that may hinder the utilisation of healthcare services through health education. The impact of promoting tetanus vaccination for pregnant women appears to go beyond the mother’s health and may have an impact on her willingness to prioritize vaccination of the child.

Our study also found that households belonging to the *Bunda* and *Mbala* ethnicities were about half as likely to have ZD children as *Pende* ethnicity in the same city. DRC is a country of great cultural diversity, with over 450 ethnic groups, and previous studies in Africa settings have demonstrated the influence of ethnicity on behaviour towards vaccination [49, 50]. It will be necessary to deepen the knowledge on this, in order to understand through a qualitative study why in these ethnic groups, children were less likely to be ZD compared to the *Pende* ethnic group.

The strength of this study lies in the collection of household data and not basing our results on routinely collected surveillance data that is known to have weaknesses [7], and the sampling method, covering all 49 health areas of the two HZ of Kikwit city in two weeks’ time. Having information from the health centres besides the households enabled us to understand the situation beyond household-level considerations, by taking into account other factors in the health system. The limitations of this study could be that DTP vaccination data were obtained from vaccination cards, which may contain errors or be difficult to read or interpret. We observed a few transcription errors (7.3%) on the vaccination cards, the date (day, month, and year) on which a specific vaccine is administered must be indicated on the card, but some vaccination cards indicate "day and month" or "month and year" or simply a tick beside the administered vaccine. These errors could be confusing for interpretation when making calculations. If the card was not available, vaccination data were collected from the mother/guardian’ recall. Several research in the same field rely on maternal recall as a method to gather data on childhood immunisation. This is because vaccination cards are less accessible at household level and vaccination records at health facilities level are inadequately maintained [8, 9, 25, 51, 52]. Nevertheless, the maternal recall of childhood immunization may be biased.

At health area level, we collected data on the availability of refrigerator, the accessibility of some avenues/villages, and the vaccine stockout during the last 12 months, but could have included other factors, such as the supervision of vaccination sites, the number of vaccination sessions during a given period (week, month), the motivation or incentive of health professionals, etc. Cluster analysis is a statistical technique and assumes no underlying knowledge of the situation or how population may behave. In other words, it is just clustering the data around a series of central points–which way it may or may not make sense, that is why we combined this technique with a multilevel regression approach not only to identify the existence of clusters but also risk factors, which made results more understandable. And this study was cross-sectional and hence, cannot define trend over time.

This study provided us with the prevalence of ZD children at a fine-scale (health area) level to enable targeted interventions to reach such children. This data can also be used in future research to assess the performance of other techniques of estimating ZD children at fine-scale prevalence [19, 53]

## Conclusion

Our findings demonstrated a high prevalence of ZD children with a substantial heterogeneity in the city and identified two hotspots within the South HZ, with one of the hotspots overlapping with health areas in North HZ. Due to sub-zonal diversity, a health zone approach to reduce ZD immunization appears limited. ZD prevalence was related to the CHWs’ home visit, to the distance of residence to a health centre and to household-level factors. Geospatial results could help in targeting priority health areas and communities for vaccination with the support of CHWs.

## Supporting information

S1 Data A QGIS-compatible geospatial file containing the results of geospatial analysis, which may be accessed using QGIS software. (QGS)
